# MDA-MET-conditioned-medium augments the chemoattractant-dependent migration of MDA-MET cells towards hFOB-conditioned medium and increases collagenase activity

**DOI:** 10.1186/s12885-017-3315-4

**Published:** 2017-05-11

**Authors:** Karis Chin-Quee, Henry J. Donahue

**Affiliations:** 1 Department of Orthopaedics and Rehabilitation, Penn State College of Medicine, Hershey, PA 17033-2391 USA; 2 Department of Biomedical Engineering, Institute of Engineering and Medicine, Virginia Commonwealth University, 601 West Main Street, Richmond, VA 23284-3067 USA

**Keywords:** Migration, Type 1 collagen fragment, Collagenase, Breast cancer, Metastasis, Pre-metastatic niche osteoblasts

## Abstract

**Background:**

Metastasis of breast cancer displays site-specificity towards bone. Recently, studies have emerged indicating that primary tumors may remotely influence creation of a pre-metastatic niche. In this study, we used human fetal osteoblastic cells and MDA-MET, a metastatic and preferentially bone homing derivative of the breast cancer cell line MDA-MB-231. We examined 1) whether secreted factors from MDA-MET cells increase generation of chemoattractants by human foetal osteoblastic cells 2) whether MDA-MET cells were responsive to these chemoattractants and 3) the identity of these chemoattractants.

**Methods:**

Human foetal osteoblastic cells were treated with conditioned medium of MDA-MET cells for 24 hours and then washed with phosphate-buffered saline. Serum-free replacement medium was conditioned by treated hFOB cells for 18 hours, before its use in *in vitro* quantification of MDA-MET migration. We also quantified collagen levels and collagenase activity in conditioned medium from human foetal osteoblastic cells.

**Results:**

Conditioned medium from human foetal osteoblastic cells that had been treated with MDA-MET-conditioned medium attracted more MDA-MET cells than hFOB cells pre-exposed to their own medium. This conditioned medium had increased collagenase activity. The addition of bacterial collagenase removed the ability of conditioned medium from human foetal osteoblastic cells to attract MDA-MET cells.

**Conclusions:**

Our data suggest that an increase in collagenase activity in osteoblastic cells induced by their exposure to breast cancer cell–secreted factors may increase their ability to attract breast cancer cells.

## Background

Approaches to cancer treatment have limited success when a tumor has metastasized. The reason for this is not currently known. While it is possible that metastasized tumors are simply too different to respond to the same approaches as primary tumors, a more likely explanation is that metastasis is never detected early enough to be treatable by methods that have proven successful for an early primary tumor.

In 2005, Kaplan et al. produced in vivo evidence that secreted factors from a primary tumor could modify its metastatic target in a pro-metastatic manner [1]. They summarized these changes by coining the term the “pre-metastatic” niche; which they predicted would appear in a tissue known to be favored by a given tumor for metastasis. Manipulation of such a niche could possibly prevent establishment of a metastasis. In the Kaplan study, fibronectin, a glycoprotein component of extracellular matrix, was shown to be increased in lungs of C57BL/6 black mice injected with conditioned medium from B16 melanoma cells; a cell line which shows a high proclivity for lung metastases. Clusters of haematopoietic cells formed in lungs following injection and preceded its colonization by B16 melanoma cells. Metastasizing melanoma cells co-localized with these clusters with 90% precision. These results suggested that changes in lung, precipitated by exposure to the conditioned medium, were pro-metastatic.

In an ex vivo study, Hiratsuka et al. [2] using B16 melanoma cells, showed that exposure to melanoma-conditioned medium caused lung tissue to undergo pro-metastatic changes. A gene array was completed revealing an increase in mRNA expression encoding matrix metalloproteinase-1 (MMP1), a collagenase which breaks the triple helix of collagen into distinct fragments. A third study also implicated the extracellular matrix as a player in the pre-metastatic niche [3]. Lysyl oxidase (LOX), an enzyme, important in collagen turnover and secreted by hypoxic breast tumor cells, was shown to accumulate at pre-metastatic sites where it caused crosslinking of collagen IV in the basement membrane. This crosslinking was thought to form a pre-metastatic niche by recruiting CD11b + myeloid cells as evidenced by the fact that LOX inhibition prevented both CD11b + cell recruitment and metastatic growth. These studies not only provide evidence of the existence of a pre-metastatic niche, in an organ which is a preferred metastatic target for a given tumor, but also suggest that agitation of the extracellular matrix is crucial to the facilitative nature of the pre-metastatic niche. The exact mechanism by which this agitation may facilitate metastasis is not known.

In 70% of patients with metastatic breast cancer, secondary metastatic tumors are present in bone tissue [4]. According to the definition of pre-metastatic niche, if such a phenomenon exists for breast cancer, bone would be a likely candidate for the establishment of this niche. This is especially true in light of the fact that bone is a hypoxic environment and LOX activity is particularly important. The bone environment in the original context of a pre-metastatic niche as described by Kaplan [1] for breast cancer has not been studied before. We sought to determine whether a pre-metastatic niche was established in bone by breast cancer secreted factors. Thus, if a pre-metastatic niche is created by circulation-borne factors secreted by a primary tumor, a cell line with a high proclivity for metastasizing to bone is likely to generate more changes that facilitate metastasis than a less bone-specific one. To examine this concept we exposed osteoblastic cells to conditioned media from breast cancer cells and non-cancerous breast epithelial cells and quantified bone metastatic characteristics. Osteoblasts, being similar to bone lining cells, were chosen for this study, since in vivo they act as an early physical defense against invading breast cancer cells.

We found that exposure to secreted factors from breast cancer cells caused bone cells to increase their attraction of breast cancer cells. Although Festuccia et al. and Mundy et al. [5, 6] have shown that osteoblast conditioned medium can increase migration of cancer cells through a collagenase related system, this is the first time this change has been shown to be inducible by tumor secretions. A study by Albini [7], found that the distinctive collagen fragments produced by collagenase from the collagen triple helix were a more powerful chemoattractant of dermal fibroblasts when compared to whole collagen. Given the finding of a pre-metastatic increase in collagenase MMP1 shown in lung tissue by Hiratsuka [2] and evidence from Mundy that both collagen and collagen fragments are a chemoattractant for MDA-MB-231 cells [6] we also examined: 1) whether collagenase activity increases in osteoblasts exposed to breast cancer cell-conditioned medium and 2) whether this could account for our observation of increased production of MDA-MET- responsive chemoattractants by hFOB cells.

## Methods

### Cell lines and culture

Cell lines used in this study were: 1) the telomerase-immortalized, non-malignant breast epithelial cell line HTERT-HME1 (HTERT; ATCC CRL-4010) [8] maintained in a specially formulated, “Mammary Epithelial Cell Basal Medium” (Clontech, Lexington, KY) containing: 2.5 ml BPE, 0.5 ml hEGF, 0.5 ml hydrocortisone, 0.5 ml GA-1000, 0.5 ml and insulin; 2) MDA-MET, the bone- selective derivative of MDA-MB-231 breast cancer cell line [9] (from Dr. Larry Suva, University of Arkansas) was maintained in high glucose DMEM medium (GIBCO, Life Technologies, Inc., Rockville, MD) supplemented with 10% FBS. 3) hFOB 1.19 (hFOB; originally from Steve Harris, Mayo Clinic), human fetal osteoblastic cells were maintained in F12/DMEM supplemented with 10% FBS and 1% penicillin-streptomycin [10] and 4) human bone marrow endothelial cells (HBMEC) immortalized with a retroviral construct containing the human papilloma virus 16 E6/E7 genes [11] were cultured in a specially formulated medium consisting of Medium 199 (Sigma-Aldrich, St. Louis, MO) supplemented with 10% FBS and 1% penicillin–streptomycin, 1% GlutaMax (Invitrogen), Endothelial Cell Growth Supplement 0.015 mg/ml Heparin 0.1 mg/ml (Sigma-Aldrich, St. Louis, MO).

MDA-MET, hFOB, HBMEC and MDA-MB-231cells were plated at 1.3 × 10^4^ cells/cm^2^ in a 100 mm dish in high glucose growth medium, as described above, until confluent (approximately 3 days). HTERT-HME1 cells were also plated in a 100 mm dish at 2.6 × 10^6^ cells/cm^2^ and allowed to reach confluency (approximately 3 days).

### Study design

hFOB cells were exposed to MDA-MET- conditioned medium or HTERT-HME1-conditioned medium for 24 h. This time period was based on a previous study in our laboratory, showing it sufficient to change the expression of osteoprotegerin [12]. hFOB cells or HBMEC cells were cultured until confluent as described in the cell culture section. Culture medium was then removed. In the case of hFOB cells, this culture medium was replaced by hFOB, MDA-MET, or HTERT-HME1 conditioned medium. Similarly, in the case of HBMEC cells, culture medium was replaced by HBMEC, MDA-MET or HTERT-HME1 conditioned medium. These conditions will be referred to as treatment medium or “medium that hFOB or HBMEC cells are exposed to”. This exposure period lasted for 24 h. Treatment medium was then removed, cells washed twice with PBS to remove all traces of serum and treatment medium replaced by DMEM/F12 serum-free cell-naïve medium. hFOB or HBMEC cells were returned to the incubator for another 18 h. hFOB-conditioned-medium or HBMEC-conditioned medium from this 18 h period was then collected and used in subsequent experiments denoted by hFOBCM [] or HBMEC [] where treatment medium is enclosed within the square brackets. Thus, hFOBCM [METCM] describes the above process where the treatment medium to which the hFOB cells were exposed was MDA-MET-conditioned medium.

### Migration assay

Migration assays were performed in a modified Boyden chamber consisting of insert wells with 8 μm pores placed in a 24-well cell culture plate. A 300 μl suspension of 1.5 × 10^5^ MDA-MET cells in serum- free medium was placed in inserts and 600 μl of hfOBCM [] or HBMECCM [] was placed in the lower well. This assay was designed to assess migration of adherent cells. Therefore, cells were counted based on the premise that these adherent cells would adhere to the underside of the transwell after passing through transwell pores. At the end of a 22-h migration period, which is in the standard range for mammalian epithelial cells, un-migrated cells that remained in the insert were aspirated and inserts placed in 225 μl of 0.05% of trypsin for 30 min. Fluorescent DNA- labeling dye CyQuant, was dissolved in lysis buffer and 60 μL of this mixture added to cells suspended in 0.5% trypsin. Corresponding fluorescence read at wavelength 480/520 nm excitation emission represented the number of cells. All migration assays used 10% FBS serum as a positive control and F12/DMEM serum-free medium as a negative control.

### Boiling

To determine whether chemoattractant-active components of hFOBCM[hFOBCM] and hFOBCM[lMETCM] were heat sensitive, we subjected them to boiling temperatures for 10 min.

### Filtration

A size exclusion filter (Millipore, Billerica, MA) was used to collect the fraction of hFOB-conditioned medium containing proteins <30 kDa. The filtration membrane allowed proteins to pass through, as filtrate was centrifuged at 10,000 rpm for 20 min. This filtrate was used in migration assays as described above.

### Bacterial collagenase assay

Activated crude bacterial (*clostridium*) collagenase enzyme, Type 2, (Worthington Biochemical Corporation Lakewood NJ 08701) was prepared at a concentration of 1 mg/ml and diluted 3 fold. 70 μL of this solution was added to hFOBCM [] in the bottom well of the Boyden chamber and used in a migration assay as described above.

### Mammalian collagenase assay

Collagenase activity was quantified using a commercially available kit (Chondrex Inc., Redmond, WA). Samples were first incubated for 70 min at 35 °C with FITC- labeled soluble collagen. If collagenase activity was present in the samples, added collagen would be digested into TC^A^ and TC^B^ collagen fragments typical of collagenases (MMP1, 8 or 13) [13]. This incubation period was followed by a denaturation step, addition of elastase, extraction and centrifugation to pellet the sample. The resulting supernatant contained collagen fragments of random size and was assessed for fluorescence. In this assay fluorescent label is undetectable in whole collagen.

### Western blot

Western blot was performed on hFOB-conditioned medium collected as previously described. In addition, it was concentrated 300% by reducing the volume by speed vacuum. 30uL was then separated by 10% sodium dodecyl sulfate-polyacrylamide electrophoresis gel and transferred to PVDF membranes. Primary antibodies used to probe the membranes were anti-Type 1 collagen 1:500 (Sigma-Aldrich, St. Louis, MO.). The membrane was also incubated with 1:2000 dilution of horseradish peroxidase-conjugated secondary antibody, incubated with working solution of ECF –plus Chemifluorescent Detection Reagents (Thermo Scientific Pierce, Rockford, IL) and imaged with a Typhoon FLA 9500 laser-based imager.

### Immunocytochemistry to detect type 1 collagen receptor UPARAP

Antibodies against urokinase-like plasminogen activator receptor associated protein (UPARAP) were obtained from Millipore (Billerica, MA.) Dapi staining indicated density of receptors relative to number of cells.

Sterile coverslips were placed in 24 well plates. 50,000 cells were sub-cultured onto cover slips over a 2 day period. MDA-MET cells were grown until confluent on cover slips. Cells were fixed in 3.7% formaldehyde at room temperature for 10 min and at −20 °C for 5 min followed by a blocking step using 5% milk for one hour. Primary antibody was diluted in 5% milk in a ratio of 1:1000 and applied to cells overnight at 4 C. Cells were washed and secondary antibody, diluted in 5% milk at 1:10,000, with a fluorescent tag was added for 30 min. The cover slip was then inverted onto a microscope slide and fixed.

#### Data analysis

All experiments were done at least in triplicate. Statistical analyses were performed by 1-way ANOVA and a post-hoc test using the Graph pad prism software or by Student’s *t* test.

## Results

**Fig. 1:**
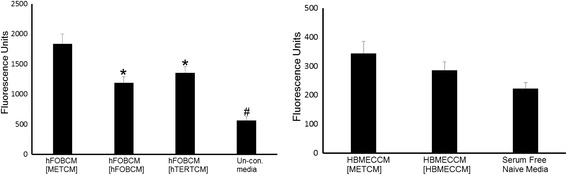
Effects of conditioned medium on hFOB cell secreted medium attraction of MDA-MET cells. *Left Panel*) hFOBCM[MDA-METCM] attracts a greater number of MDA-MET cells than either hFOBCM[hFOBCM] or hFOBCM[HTERT-HME1CM]. Conditioned medium was removed from confluent hFOB cells which had been exposed over a 24 h period to conditioned medium from confluent cells of the following lines: MDA-MET(METCM), hFOB (hFOBCM) HTERT-HME1 (HTERTCM) or un-conditioned medium. The number of migrating cells is directly proportional to fluorescence and represented as fluorescence on y axis *n* = 18–24, * *p* < 0.05 vs hFOBCM[METCM]; # *p* < 0.05 vs all other groups. *Right Panel*) There is no difference between the number of cells attracted to HBMECCM[MDA-METCM] compared to the number attracted to HBMECCM[HTERT-HME1CM] or HBMEC[HBMECCM]. Conditioned medium was removed from confluent HBMEC cells which had been exposed over a 24 h period to conditioned medium from confluent cells of the following lines: MDA-MET(METCM), HBMEC(HBMECCM) HTERT-HME1 (HTERTCM) or un-conditioned medium. The number of cells is directly proportional to fluorescence and represented as fluorescence on y axis *n* = 9

To address how exposure to MDA-MET conditioned medium would affect the ability of hFOB cells to attract MDA-MET cells, we collected hFOB conditioned medium under several conditions, as described in methods, and performed migration assays. Migration assays revealed that MDA-MET cells migrated towards hFOBCM [METCM] (conditioned medium from hFOB cells which had been previously exposed to conditioned medium from MDA-MET cells) to a greater degree than towards hFOBCM [hFOBCM](conditioned medium from hFOB cells which had been previously exposed to conditioned medium from hFOB cells), hFOBCM [HTERTCM] (conditioned medium from hFOB cells which had previously exposed to conditioned medium from HTERT-HME1 cells) or unconditioned medium (Fig. 1 *Left Panel*). Furthermore, MDA-MET cells migrated more towards hFOBCM [hFOBMCM], and hFOBCM [HTERTCM] than to unconditioned medium. In contrast, when HBMEC cells were used instead of hFOB, there was no difference among groups in the number of MDA-MET cells migrating towards HBMEC-conditioned medium, i.e. an equal number of MDA-MET cells migrated towards HBMECCM [HBMECCM], HBMECCM[METCM] and HBMECCM[HTERTCM] respectively (Fig. 1 *Right Panel*).

### Effect of boiling and size exclusion filtration

**Fig. 2:**
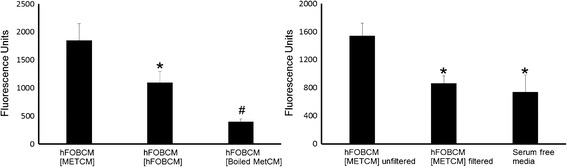
The ability of conditioned medium from hFOB cells exposed to either hFOB- conditioned medium or MDA-MET-conditioned medium can be altered by physical processing. *Left Panel*) hFOBCM[hFOBCM] and hFOBCM[MDA-METCM] lose the capacity to attract MDA-MET cells when subjected to boiling temperatures. hFOBCM[MDA-METCM] was boiled for 10 min and used in a migration assay following the identical protocol as in Fig. 1. *n* = 6, * *p* < 0.05 vs hFOBCM [METCM]; # *p* < 0.05 vs all other groups. *Right Panel*) The fraction of hFOBCM[hFOBCM] or hFOBCM[MDA-METCM] <30 kDa (filtered) did not attract MDA-MET cells. hFOBCM[hFOBCM or hFOBCM[MDA-METCM] were filtered using size exclusion filters of a membrane pore size which allowed conditioned medium components <30 kDa to pass through (filtrate). The filtrate was then used in the migration assay following the protocol described in Fig. 1. *n* = 12,* *p* > 0.05 vs hFOBCM [METCM] unfiltered

We next examined whether components in hFOBCM [METCM] that attracted MDA-MET cells in our migration assay were proteinaceous. To denature proteins and remove higher order protein structure, we first brought hFOBCM [METCM] or hFOB [hFOBCM] to boiling temperature for 10 min. Boiling hFOBCM [METCM] significantly decreased its ability to attract MDA-MET suggesting components that attract MDA-MET are proteins (Fig. 2 *Left Panel*). We next used a size exclusion filter to collect the fraction of hFOB-conditioned medium containing proteins <30 kDa in size representing chemokines and other small peptides. Filtered hFOBCM [MDA-METCM], which contains molecules <30kDA in size (including chemokines) but not larger molecules, attracted significantly fewer MDA-MET relative to unfiltered hFOBCM [MDA-METCM] (Fig. 2 *Right Panel*). This suggests that protein molecules larger than 30kDA contributed to chemoattractant properties of hFOBCM [MDA-METCM].

### Presence of type 1 collagen and effect of addition of bacterial collagenase

**Fig. 3:**
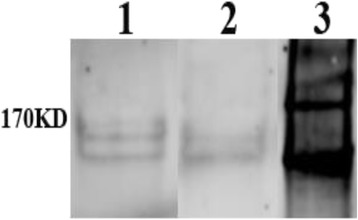
hFOBCM[MDA-METCM] (lane 1) hFOBCM[hFOBMCM] (lane 2) have detectable Type 1 collagen after 18 h conditioning period by hFOB cells. Rat tail collagen (lane 3) acts as a positive control for Type 1 Collagen antibody in a western blot analysis. This image is representative of 4 separate gels. Relevant lanes were cut out of a larger gel with more lanes

**Fig. 4:**
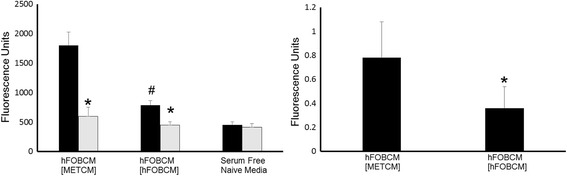
Effect of added bacterial collagenase and endogenous collagenase. *Left Lane*) Bacterial collagenase decreased the ability of hFOBCM[hFOBCM] and hFOBCM[MDA-METCM] to attract MDA-MET cells. 1 mg of bacterial *(clostridium histolyticum*) collagenase enzyme extract in lyophilized powder was added at a ratio of 1 mg/3 mls. Conditioned medium with (*gray bars*) or without (*black bars*) collagenase was used in migration assays by placing it in the bottom well of the migration chamber. *n* = 12, *p < 0.05 vs no collagenase in same group. # *p* < 0.05 vs no collagenase hFOBCM [METCM]. *Right Lane*) Collagenase activity is greater in hFOBCM[MDA-METCM] than in hFOBCM[hFOBCM]. FITC-labeled collagen was incubated with either hFOBCM[MDA-METCM] or hFOBCM[hFOBCM]. The extent of the breakdown of collagen carried out by conditioned medium samples was measured by the presence of fluorescent breakdown products in the supernatant after a centrifugation step. Intact, un-degraded collagen formed the pellet. *n* = 8, *p < 0.01

Since type 1collagen is a protein molecule greater than 30kDA in size, fragments of which attract breast cancer cells and would be abundant in the microenvironment of breast cancer cells activating bone resorption [6], we examined whether Type 1 collagen fragments might contribute to chemoattractant properties of hFOBCM [METCM]. We first demonstrated by western blot analyses that type 1 collagen is present in both hFOBCM [MDA-METCM] and hFOB [hFOBCM] (Fig. 3). We next demonstrated that adding bacterial collagenase, which produces small fragments of collagen which have been shown to lack chemoattractant power [7], to hFOBCM [METCM] and hFOB [hFOBCM] reduced the ability of both media to attract MDA-MET cells (Fig. 4 *Left Panel*). This suggests that both conditioned media contain type 1 collagen that may attract MDA-MET.

### Detection of mammalian collagenase activity

To examine why hFOBCM [MDA-METCM] attracts more MDA-MET cells than hFOB [hFOBCM], both of which contain Type 1 collagen at similar levels, we examined collagenase activity in the two media. Collagenase activity was nearly two-fold greater in hFOBCM [MDA-METCM] than hFOB [hFOBCM] (Fig. 4 *Right Panel*). Thus, while both hFOBCM [METCM] and hFOB [hFOBCM] contain similar amounts of Type 1 collagen, hFOBCM [MDA-METCM] contains more collagenase activity. This suggests that Type 1 collagen fragments, larger than those produced by exposure to bacterial collagenase, contribute to the ability of hFOBCM [MDA-METCM] to attract MDA-MET.

### UPARAP receptor is present on MDA-MET cells

**Fig. 5:**
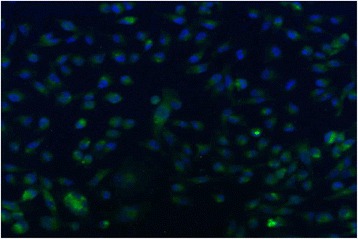
Urokinase-type plasminogen activator receptor assocated protein (UPARAP) is expressed in MDA-MET cells. Antibody for UPARAP was diluted at a ratio of 1:100 and used to perform immunocytochemistry on MDA-MET cells fixed in acetone. The presence of UPARAP is indicated by green stain while DAPI (1:10,000) stained cell nuclei appear blue

In order to examine whether MDA-MET cells had the capacity to respond to collagen fragments we performed Immunocytochemistry on MDA-MET cells testing for the presence of urokinase plasminogen activator receptor- associated protein, UPARAP, the only receptor shown to be receptive to Type 1 collagen fragments [14]. MDA-MET cells tested positive for the presence of UPARAP receptor (Fig. 5).

## Discussion

Our data suggest that, in response to exposure to secretions from breast cancer cells, osteoblasts attract more breast cancer cells than occurs without this exposure. We have also provided evidence that secretions of normal breast epithelial cells do not cause osteoblastic cells to secrete factors that attract breast cancer cells. Furthermore, changes in hFOB cells that were associated with an increased production of chemoattractants were due to cellular secretions only; without requiring the presence of the MDA-MET cells. This represents a distinction from previous studies which have explored the interaction between bone and breast cancer cells; many of which are co-culture based [15] [6] [12].

Two previous studies explored changes in osteoblasts in response to conditioned medium from MDA-MB-231 cells. In the first, intercellular adhesions were compromised which would point to a possible reduced ability to defend against invasion through the paracellular route [16]. In the second, an increase in chemokines in MDA-MB-231 conditioned medium suggested that these chemokines could contribute to chemoattraction [17]. In these two studies, whether these changes were pro-metastatic was not examined. However, osteoblastic generation of factors that result in chemoattraction suggests that these cells contribute to a pro-metastatic environment. Hiratsuka and Kaplan [1, 2] also demonstrated that chemoattractants were generated by exposure of normal tissue to tumor-secreted factors. However, these chemoattractants were ligands for receptors causing migration of haematopoietic cells and neutrophils respectively.

By using a size-exclusion filter we showed that there was no chemoattractant activity in the fraction containing compounds smaller than 30 kDa. This result suggests that chemokines, which are between 8 and 10 kDa in size [18] were not involved. Rejection of chemokines as a candidate for the identity of chemoattractants responsible for attraction of MDA-MET cells in our study led us to our hypothesis that collagen fragments and not chemokines were acting as chemoattractants, and our results support this hypothesis. Furthermore, Mundy et al [6] demonstrated that TCA and TCB collagen fragments, which would result from collagenase breakdown of Type 1 collagen, act as chemoattractants to MDA-MB-231 cells, the parent line of the MDA-MET cells. Taken together these results suggest that increased collagenase in hFOBCM[METCM] results in increased collagen fragments which in turn increases chemoattraction. This finding is supported by a study by Festuccia et al. [5] showing that osteoblast conditioned medium, possibly through TGF beta, can increase migration of cancer cells using a collagenase-related mechanism.

Our study design, which employed a Boyden chamber, shows a simple chemoattractant and migratory cell relationship. The Kaplan and Hiratsuka studies and others (reviewed in [19]) required the presence of bone-marrow derived cells to create the niche. Here we show that, in vitro, pro-metastatic changes occur without intermediates suggesting tumor cells alone have the potential affect osteoblastic cells.

Interestingly, MMP1, an enzyme which creates collagen fragments found to be the chemoattractant in the present study, and whose expression was up-regulated in liver tissue in response to B16 melanoma secretions [2], has been implicated as an early indicator of metastasis; preceding the establishment of a successfully metastasized tumor. Barkan et al showed that dormant metastasized cells could be aroused and coaxed into colonizing their previously undisturbed habitat due to effects of the collagenase MMP1 [20]. In Barkan’s study, the trigger for this increase in collagenase activity leading to changes in behavior of previously dormant cells was not identified. Since these are dormant cells and not considered to be a malignant tumor, they can also be considered to constitute a pre-metastatic niche; although they are usually cited in support of the cancer stem cell theory(reviewed in [21]) Thus, the Barkan study is yet another study where agitation of the extracellular matrix at the metastatic target was shown to be associated with facilitating metastasis.

## Conclusions

In conclusion, we have shown evidence that when osteoblasts are exposed to brest cancer cell conditioned medium they attract more breast cancer cells than if they are exposed only to their own medium. Additonaly, this might be due to an increased production of collagen fragments which act as chemoattractants to breast cancer cells. It is imoportamt to emphasize, however, that future studies will need to confirm these initial findings.

## Abbreviations

CM: conditioned medium
HBMEC: human bone marrow endothelial cells
hFOB: human fetal osteoblastic cells
HTERT-HME1: human telomerase reverse transcriptase transfected human mammary epithelial cells
LOX: lysyl oxidase
MMP-1: matrix metalloproteinase-1
